# Symphysis Pubis Osteomyelitis: An Uncommon Complication after Robotic Assisted Radical Prostatectomy—Case Description with Literature Review

**DOI:** 10.1155/2018/5648970

**Published:** 2018-02-13

**Authors:** Jad A. Degheili, Mazen M. Mansour, Rami W. Nasr

**Affiliations:** Division of Urology and Renal Transplantation, Department of Surgery, American University of Beirut Medical Center, Riad El-Solh, Beirut 1107 2020, Lebanon

## Abstract

Prostate cancer is the most common solid cancer among American men. Although there are various modalities for treatment, including radical prostatectomy among many others, the former is, nevertheless, not without any accompanied complications. Other than the well-known surgical complications such as erectile dysfunction, urinary incontinence, and voiding dysfunction, osteomyelitis of the symphysis pubis is not a well-understood complication, with various hypotheses explaining its pathogenesis. Although osteomyelitis of the pubis symphysis has been reported after endoscopic urological procedures such as transurethral resection of the prostate, it has rarely been reported after robotic surgeries. We hereby report, to the best of our knowledge, the first osteomyelitis of the pubis symphysis, after robotic prostatectomy, in a patient with prostate cancer and no previous radiation therapy.

## 1. Introduction

An estimated number of 161,360 newly diagnosed cases of prostate cancer and 26,730 death from prostate cancer are reported among North Americans in 2017 [1]. About 1 in 7 men will be diagnosed with prostate cancer during their lifetime, and usually it is a disease of the elderly population [2]. Prostate cancer is the third leading cause of death following lung and colorectal cancer, respectively. Currently 3 million survivors from prostate cancer are currently alive [2].

A number of guidelines exist that address prostate cancer treatment, depending on the extent of the disease, with multiple treatment options available, including surgical resection, that is, radical prostatectomy, radiation therapy, or even some other newer forms of energy ablative therapies including cryotherapy [3, 4]. Along with these treatment modalities, a spectrum of side effects does exist, most importantly voiding dysfunction, urinary incontinence, and erectile dysfunction [3]. Infectious osteomyelitis (OM) of the pubis symphysis after radical prostatectomy is a rare event, often debilitating and requiring long-term treatment and occasionally surgical debridement.

The current body of literature is scarce including only few sporadic case reports of infectious OM after urological procedures, especially following robotic assisted radical prostatectomy (RARP). We hereby present the first case, of infectious pubis symphysitis following RARP, in a patient without any radiation therapy exposure.

## 2. Case Description

A 68-year-old diabetic man who has been followed up for his elevated prostate specific antigen (PSA) level has underwent a transrectal ultrasound biopsy of the prostate, confirming the diagnosis of a Gleason 7 (4 + 3) prostate cancer. Metastatic workup including PET/CT choline scan was negative. A RARP with bilateral pelvic lymph nodes dissection was performed six weeks later. Postoperative course was uneventful, and he was discharged home two days later, to follow up after one week for removal of his indwelling catheter. Pathology revealed a localized prostate cancer disease with negative lymph node involvement and negative surgical margins, consistent with a pT2bN0 disease. No adjuvant radiation therapy was needed; patient was advised to repeat his PSA level every 3 months, consecutively.

**Figure 1:**
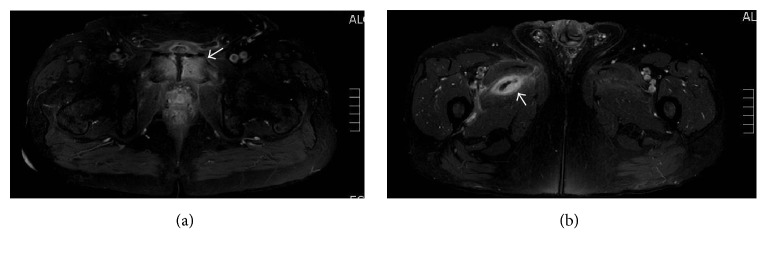
MRI axial imaging of the pelvis and thighs using the SPIR (Spectral Presaturation with Inversion Recovery) hybrid fat suppression technique. (a) Increased bone marrow signal with enhancement involving the symphysis pubis (arrow) consistent with osteomyelitis and moderate surrounding soft tissue edema. (b) A multiloculated fluid collection (arrow), with rim enhancement, within the right adductor muscle, measuring 3.8*∗*2.0*∗*7.7 cm.

One month later, the patient presented back with a two-week complaint of pelvic and suprapubic pain, waddling gait, and occasional low grade fever of 38.5°C. Laboratory workup showed leukocytosis of 11,600/mm^3^ and CRP level of 179 mg/L. Because of his severe suprapubic pain, a MRI of the pelvis with gadolinium was requested, revealing the presence of increased bone marrow signal with enhancement involving the symphysis pubis, consisting with osteomyelitis (Figure 1(a)). A 2.1 *∗* 1.0 cm fluid collection, with enhancing rim, was also identified, anterior to the pubis symphysis, in keeping with an abscess, and multiple other fluid collections within the adductor muscles bilaterally, largest on the right side, measuring 3.8 *∗* 2.0 *∗* 7.7 cm (Figure 1(b)).

Under CT-guidance, drainage of the right adductor muscle collection was performed, and patient was started on broad-spectrum antibiotics, including Vancomycin and Meropenem, pending fluid cultures, which later grew enterococcus species. His CRP level dropped to 43.7 mg/L.

Seven days after drainage, the patient witnessed an increase in his pubic pain. A follow-up enhanced MRI scan revealed no significant change in osteomyelitis of the symphysis pubis, with significant decrease in the previously described right adductor brevis muscle abscess, now measuring 2.5 *∗* 2.0 *∗* 2.7 cm; however, a new fluid collection was noted in the anterior aspect of the right gracilis muscle, compatible with an abscess. Repeated CRP level increased again to 74.3 mg/L. The antibiotics were then changed to tazobactam/piperacillin, with mild improvement in his symptoms.

**Figure 2:**
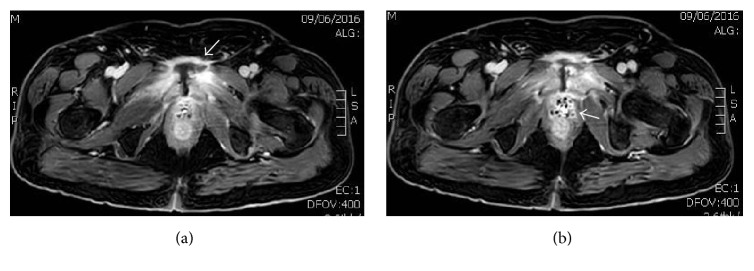
MRI axial imaging of the abdomen and pelvis pre- and postadministration of intravenous Dotarem® contrast. (a) Further distention of the symphysis pubis joint secondary to an increase in size of an abscess within it (arrow), measuring 3.8*∗*1.8 cm, compared to previous measurement of 2.0*∗*1.5 cm. (b) Apparent pocket of air around the prostatic urethra (arrow) likely representing susceptibility artifact from surgical sutures rather than actual gas bubbles.

**Figure 3:**
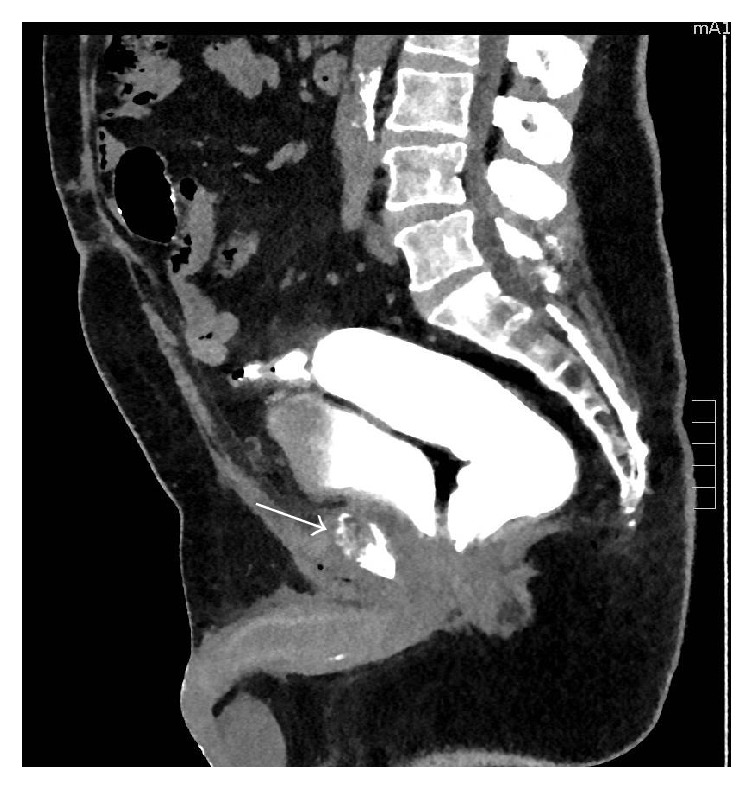
CT sagittal view of the abdomen and pelvis after administration of rectal enema, following the retrograde urethrogram. No evidence of contrast leakage from the bladder, urethra, or rectum is seen. No air bubbles are seen in the retropubic area in the location of the suture material that was reported on the previous MRI, confirming that the appearance on the MRI was related to surgical sutures, rather than leak. The bony abnormality in the symphysis pubis is also demonstrated in the view (arrow).

Two weeks later, a follow-up MRI scan revealed a worsening of the patient's symphysis pubis septic arthritis, with increase in size of the parasymphysial abscess and surrounding inflammation (Figure 2(a)). A new finding on MRI showed, this time, the presence of gas pockets surrounding the prostatic urethra, which likely represents a susceptibility artifact from surgical sutures rather than gas bubbles (Figure 2(b)). These appear more conspicuous than on prior MRI studies, which is mostly due to the different types of postcontrast imaging sequence used in the current scan. Just not to miss any fistulous tract originating from the rectum or urethra, a retrograde urethrography with a barium enema, followed by a CT scan, was then performed. No evidence of contrast leakage from the bladder, urethra, or rectum was noted (Figure 3).

The orthopedic team recommended surgical debridement of the symphysis pubis and the necrotic bone around it. Following that, excessive irrigation was performed with application of vancomycin-impregnated beads within the area of the excised bone. The right-sided adductor brevis abscess was also drained, intra-op, and a large bore Hemovac® drain was inserted. The pathology of the debrided bony tissue turned out to be acute and chronic osteomyelitis. Afterwards, patient clinically improved and was resumed on intravenous vancomycin and Tazocin. Ten days later, patient was discharged home on Rifampicin and Ciprofloxacin for a total of 12-week course. Two months later, his condition significantly improved, and he resumed his normal ambulatory activities. A Leukoscan showed no evidence of radiotracer avid infectious process, with symptoms free, six months later.

## 3. Discussion

The incidence of prostate cancer is subject to variations among various geographical locations; a value up to 50-fold difference in rate has been reported among various international populations [5]. Whilst its incidence is high in Western Europe and North America, it is however low in Asian and Middle Eastern countries [6]. Based on Globocan 2012 data, a lower age-standardized incidence rate (ASIR) of prostate cancer was reported from the Middle East and North Africa (MENA) region, compared to Western countries [7]. Lebanon, for example, had an increase in ASIR for prostate cancer from 29.9 per 100,000 to 39.2 per 100,000, in 2008 [8]. Almost similar figure was also reported in 2012, 37.2 per 100,000 [9].

On the other hand, the age-standardized mortality rate (ASMR) in 2012, in our country, was reported to be 17.1/100,000, compared to 9.8 and 5.0/100,000 in USA and Japan, respectively [7]. This is expected to rise, even further, based on future projection models. In Lebanon, the prostate cancer incidence is expected to be 64 per 100,000 by 2018 [8], with overall mortality rate from prostate cancer, in the MENA region, increasing from 15,422 in 2012, to 19,681 deaths in 2020 [7].

Multimodality treatment approaches for prostate cancer are available and include active surveillance, radical prostatectomy, external beam radiation, androgen deprivation therapy, brachytherapy, or even high intensity frequency ultrasound therapy. For those treated with surgery, especially those with localized disease, robotic assisted surgery is one of the innovative surgical approaches, nowadays used [10]. Commonly reported side effects, following prostate cancer surgeries, include erectile dysfunction, voiding dysfunction, and urinary incontinence [3]. Nevertheless, few patients can experience very rare and poorly understood complications, for which osteomyelitis of the symphysis pubis, after robotic assisted radical prostatectomy, is one of them.

Osteomyelitis of the symphysis pubis is a rare entity accounting for less than 1% of all cases of osteomyelitis [11]. Ross and Hu had reviewed 100 cases of pubic symphysis septic arthritis and had reported that such rare entity is not specific to any age group, with age is ranging from 7 to 86 years [12]. The presenting signs and symptoms are mainly pubic pain, antalgic gait, pain with hip motion, and occasional presence of fever. Postoperative pain after radical prostatectomy is expected; nevertheless, pain lasting for more than 6 months is not usual and may suggest other etiologies [13].

Osteomyelitis of the symphysis pubis has been reported after renal transplantation [14], inguinal herniorrhaphy [15, 16], and procedures for urinary stress incontinence such as tension-free vaginal/ transobturator taping [17]. It has also been reported following vaginal birth delivery [18], cardiac catheterization [19], chronic indwelling catheterizations [20], or even after abuse of parenteral drugs [21]. More pertinent osteomyelitis of the symphysis pubis has also been reported after transurethral resection of the prostate or even incision of the bladder neck, in a previously irradiated pelvis [22].

Several theories lie behind the pathogenesis of pubic bone osteomyelitis. This includes infection, trauma, secondary to haematogenous spread, or complex regional pain syndrome [23]. The most common pathogen causing pubic symphysis osteomyelitis is* Staphylococcus aureus*. However, other organisms such as* Pseudomonas aeruginosa*,* Escherichia coli*,* Enterococcus* species,* Mycobacterium tuberculosis*, and* Salmonella* species have also been reported in the literature [24].

There have been several reports of pubic bone osteomyelitis secondary to pubosymphyseal fistula, in patients who underwent radical prostatectomy, followed by adjuvant radiation therapy. Matsushita et al. reported 12 patients, from two centers over an 11-year period, who developed a pubovesical fistula, following treatment of prostate cancer [25]. All those patients had radiation therapy either as the primary treatment or as a salvage therapy and had subsequently developed bladder neck contracture. The median interval to develop pubovesical fistula was 37 months, after treatment for bladder neck contracture. Broad-spectrum antibiotics were initiated in all patients, and only one patient had resolution of his osteomyelitis. The remaining patients either necessitated diversion of their urinary tract or insertion of bilateral percutaneous nephrostomies, to achieve resolution of symptoms [25]. Despite such a small sample size, the long time frame, extending over 11 years, reflects the rarity of this disease and as such the absence of any existing guidelines, for treating those fistulas.

Pelvic bony pain and gait instability, after radical prostatectomy, reflect multiple bone etiologies, to contemplate in our differential diagnosis. Other than osteomyelitis of the pubic symphysis, pelvic insufficiency fracture (PIF), osteonecrosis (ON), and osteitis pubis (OP) are among the others to consider [13]. Osteitis pubis is usually mistaken by osteomyelitis. It is defined by a painful inflammatory process resulting in bony destruction of the margins of the symphysis pubis [23]. Nevertheless, OP is a self-limiting process, treated conservatively by anti-inflammatory drugs. The main difference between osteitis pubis and osteomyelitis is the negative culture on biopsy [26]. Noteworthy here is that delay in diagnosis or treatment of symphysis pubis osteomyelitis can further manifest as bilateral thigh pain and adductor muscle abscesses, necessitating percutaneous drainage [24, 27].

As part of pain relief, pubic bone resection has been shown to provide immediate and sustained improvement in pain, along with the long course of antibiotics administered [28]. In a cohort of 16 patients, a statistically significant decrease in the median pain intensity score was noted, over a median follow-up of 9.4 months, after performing pubic bone resection (5.5 versus 0; *P* = 0.0005).

Suturing of the dorsal venous complex (DVC), during the robotic radical prostatectomy procedure, using a type of needle called the V-lock suture needle, and then fixing it at the level of the pubic bone,, has been postulated to be thecause for osteomyelitis. In addition, repeated needle injury to the pubic symphysis, during the urethral vesical anastomosis, may inadvertently cause contiguous-focus osteomyelitis, as well. Yet, the possibility of a rarer route for the etiology of the pubic bone osteomyelitis cannot be excluded, given the negative urine culture at time of surgery. Our patient's complication soon occurred after surgery, rather than several months later, as most reports usually do highlight. Whether this hypothesis, secondary to our surgical technique per se, is valid, it may still be debated. As such, urologists are advised for caution when suturing the anterior portion of the urethrovesical anastomosis to avoid any injury to the pubic mantle.

Very few case reports have discussed the bony complications after radical prostatectomy and radiation therapy [29–32]. Yet this complication is rarely deliberated in literature after robotic radical prostatectomy [13]. Hereby, we present our case, which might probably be the first case of infectious pubis osteomyelitis, after RARP, without adjuvant radiation.

## 4. Conclusion

Osteomyelitis of the symphysis pubis is a rare condition, very uncommon to occur after radical prostatectomy. It can be often missed due to vague nonspecific symptoms. Clinicians should always have high index of suspicion for this exceptional complication, especially in patients presenting with pelvic discomfort after prostate surgeries. Treatment of choice should be aggressive antibiotics' initiation and early surgical debridement.

## Abbreviations

PCa: Prostate cancer
OM: Osteomyelitis
RARP: Robotic assisted radical prostatectomy
PSA: Prostate specific antigen
CRP: C-reactive protein
MRI: Magnetic resonance imaging
CT: Computed tomography
PIF: Pelvic insufficiency fracture
ON: Osteonecrosis
OP: Osteitis pubis
DVC: Dorsal venous complex.
